# Atypical burkitt's lymphoma transforming from follicular lymphoma

**DOI:** 10.1186/1746-1596-6-63

**Published:** 2011-07-08

**Authors:** Yu Y Hwang, Florence Loong, Lap P Chung, Chor S Chim

**Affiliations:** 1 Department of Medicine, Queen Mary Hospital, University of Hong Kong, Pokfulam, Hong Kong; 2 Department of Pathology, Queen Mary Hospital, University of Hong Kong, Pokfulam, Hong Kong

## Abstract

Amongst follicular lymphoma that transforms into a high-grade lymphoma, majority are diffuse large B cell lymphoma. Here we reported a rare atypical Burkitt's lymphoma transformation from an asymptomatic follicular lymphoma. Lymph node biopsy showed a composite lymphoma with infiltration of the inter-follicular areas by high grade small non-cleaved lymphoma cells amongst neoplastic follicles. Moreover, FISH and molecular genetic study confirmed concomitant MYC translocations and t(14;18) in the high-grade component, thereby suggesting the transformation of atypical Burkitt's lymphoma from an undiagnosed antecedent follicular lymphoma. The disease followed an aggressive clinical course, terminating in refractory disease 13 months after diagnosis. This is followed by a comprehensive review of the literature on lymphoma transformations from underlying follicular lymphoma after acquisition of MYC translocation, using Burkitt's lymphoma, follicular lymphoma, transformation and MYC translocations as keywords.

## 

Follicular lymphoma (FL) is a low grade lymphoproliferative disease which frequently presents with generalized lymphadenopathy and frequent bone marrow involvement. The lymphoma arises from germinal centre lymphocytes with three histological grades based on the number of centroblasts in the neoplastic follicles. The pathogenesis of disease is due to the overexpression of the anti-apoptotic *Bcl-2 *associated with t(14;18), which juxtaposes *BCL-2 *gene on chromosome 18 to the enhancer of the immunoglobulin heavy chain gene (IgH) locus on chromosome 14. Moreover, as in their normal counterparts, the FL cells have on-going somatic hypermutations [1], which are implicated as a possible mechanism of high grade transformation [2].

Up to 30% of patients with FL had high grade transformation of their disease [3]. In a study of 38 patients with histological progression from antecedent follicular lymphoma, the most common histology was diffuse large cell (68%), followed by diffuse mixed (21%), and small non-cleaved cell histology only constituted 5% of transformations from FL [4]. On the other hand, in patients presenting with atypical Burkitt's lymphoma, about 25% were transformations from an antecedent FL [5].

MYC, located at chromosome 8q24, is a transcription factor involved in various translocations [t(8;14), t(8;22), t(2;8)], leading to its dysregulation and hence carcinogenesis. Acquisition of MYC translocation was reported in some high grade transformation of FL. Here we presented a patient with atypical Burkitt's lymphoma showing evidence of transformation from an undiagnosed antecedent FL. This is followed by a review of the literature on lymphoma transformations from underlying FL after acquisition of MYC translocation.

## Report of a case

**Figure 1:**
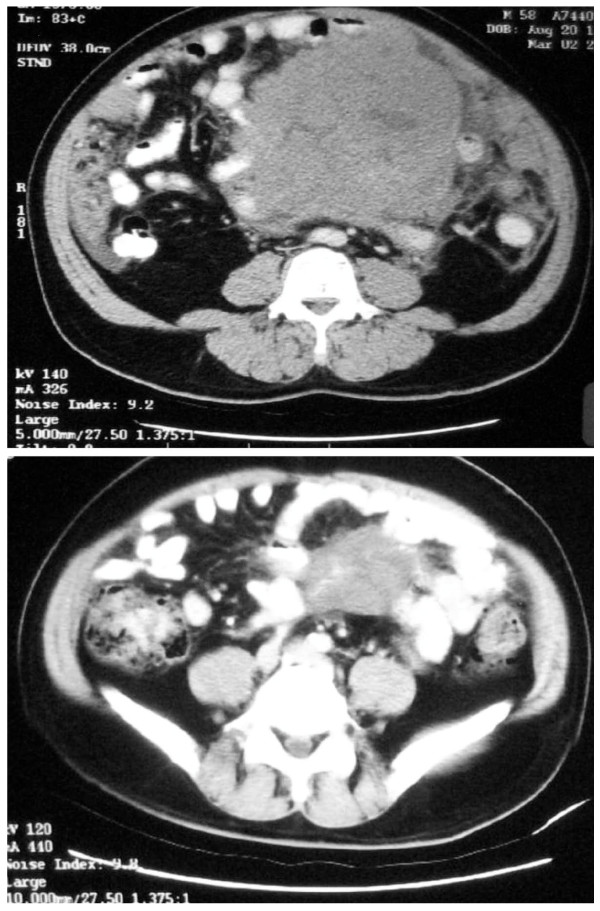
**(A) CT abdomen showed a huge enhancing lobulated mass of 15.5 cm in diameter in the central abdomen before chemotherapy**. (B) Reassessment CT abdomen after chemotherapy showed persistent central abdominal mass measured 7 cm in diameter.

A 58 year-old man with good past health, presented with progressive abdominal swelling for 4 months prior to admission. He did not have any constitutional symptoms. Physical examination showed a grossly distended abdomen and a right submandibular lymph node measuring two centimeter (cm). Computer tomography (CT) of the abdomen showed a huge contrast enhancing lobulated mass of 15.5 cm in diameter in the central abdomen (Figure 1A), infiltrating the jejunum and ascending colon. In addition to the soft tissue deposits on anterior abdominal wall, multiple enlarged mesenteric lymph nodes and ascites were noted. Biopsy of the submandibular lymph node showed composite lymphoma with both grade 1 FL and atypical Burkitt's lymphoma. On the other hand, abdominal lymph node biopsy showed diffuse infiltration by atypical Burkitt's lymphoma cells. Polymerase chain reaction (PCR) for t(14;18) was positive while Epstein-Barr virus encoded RNA (EBER) was negative in both the submandibular and abdominal lymph node specimens. The serum lactate dehydrogenase (LDH) was 2732 U/L (Normal < 401 U/L). The patient tested negative for Human Immunodeficiency Virus (HIV) serology. Bone marrow examination did not show lymphoma involvement. He had bulky Ann Arbor Stage III_A _atypical Burkitt's lymphoma transforming from underlying FL, and belonged to age-adjusted International Prognostic Index high intermediate risk.

He received intravenous cyclophosphamide and vincristine together with oral prednisolone. After initial cytoreduction, he was induced with Stanford V regimen [6]. He achieved only a partial response after a total of 5 cycles of Stanford regimen. Subsequent reassessment CT scan showed that while the other small intra-abdominal deposits reduced in size remarkably, there was only moderate shrinkage of the major central abdominal mass, which still measured seven cm in diameter (Figure 1B). The chemotherapy was therefore switched to ifosfamide, etoposide and high dose cytarabine (IVAC) [7]. The disease remained refractory despite two cycles of IVAC with development of new hepatic lesions, progressive enlargement of central abdominal mass and serum LDH rising to more than 5000 U/L. This was further complicated by gastrointestinal bleeding but the bleeding source could not be localized despite upper endoscopy and colonoscopy. He was further salvaged with fludaradine, mitoxantrone and dexamethasone but he succumbed finally to refractory lymphoma.

## Pathology

**Figure 2:**
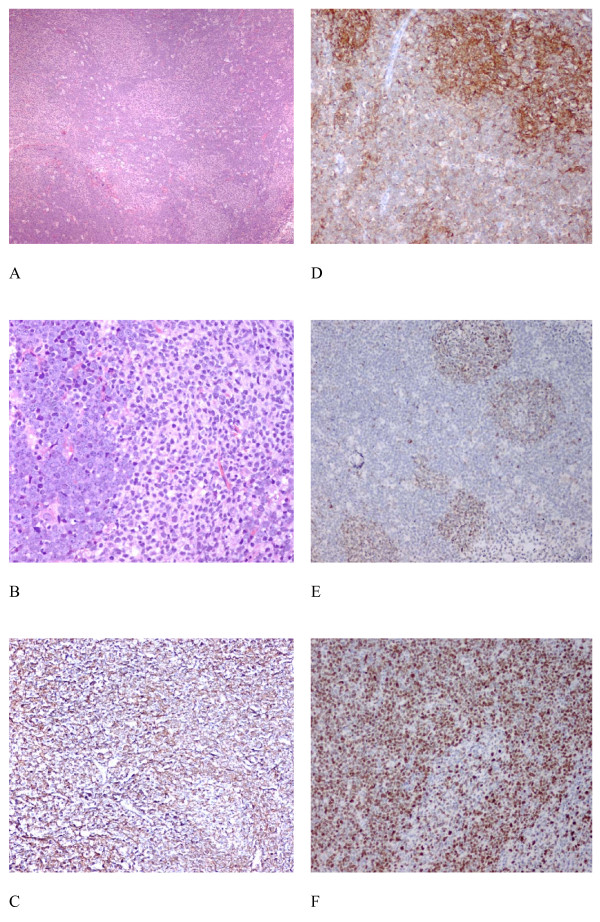
**Histological features of the submandibular lymph node**. The histology showed neoplastic follicles and a high grade interfollicular lymphomatous infiltrate featuring starry sky pattern (Figure 2A, × 40 H&E; Figure 2B, × 200 H&E) with frequent mitotic and apoptotic bodies. Immunohistochemical study showed that CD20 is strongly expressed in both the follicular and diffuse components (Figure 2C, H&E × 100), and CD10 is strongly expressed in the follicular component while it is only weakly positive in the interfollicular high grade component (Figure 2D, ×100). The neoplastic follicles are positive for BCL2 (Figure 2E, × 100) and around 95% MIB1 immuno-reactivity is noted in the interfollicular high grade lymphoma infiltrate (Figure 2F, × 100).

Excision biopsy of the submandibular lymph node (Figure 2) showed composite histology with neoplastic follicles, and a high grade interfollicular lymphomatous infiltrate featuring starry sky pattern (Figure 2A and 2B) with frequent mitotic and apoptotic bodies. Immunohistochemical study showed that CD20 was positive in the follicular, inter-follicular and diffuse components whereas CD10 was strongly expressed by the follicular component but was only weakly positive in the interfollicular high grade component (Figure 2C and 2D). The neoplastic follicles, but not the high-grade interfollicular Burkitt's lymphoma cells, showed aberrant expression of BCL2 (Figure 2E). BCL6 was expressed in both the neoplastic follicles and the atypical Burkitt's lymphoma cells. The interfollicular high grade lymphoma infiltrate showed > 95% MIB1 immuno-reactivity (Figure 2F) while the neoplastic follicles displayed MIB1 immuno-reactivity in 20%-30% cells only. The histological features were consistent with an atypical Burkitt's lymphoma transforming from grade 1 FL.

**Figure 3:**
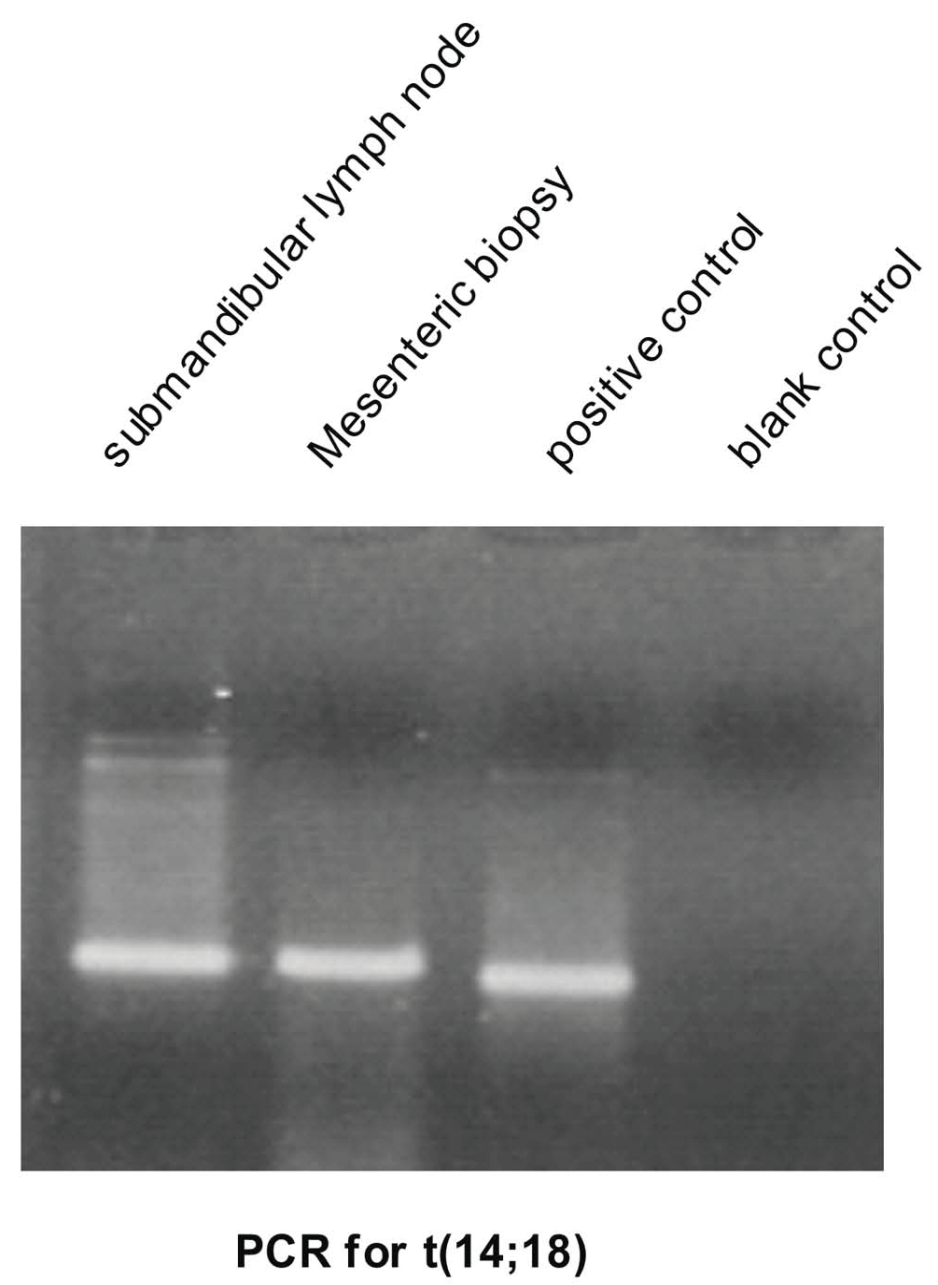
**PCR for t(14;18) showed positive amplifcation of the same size in both the submandibular and abdominal lymph node**.

**Figure 4:**
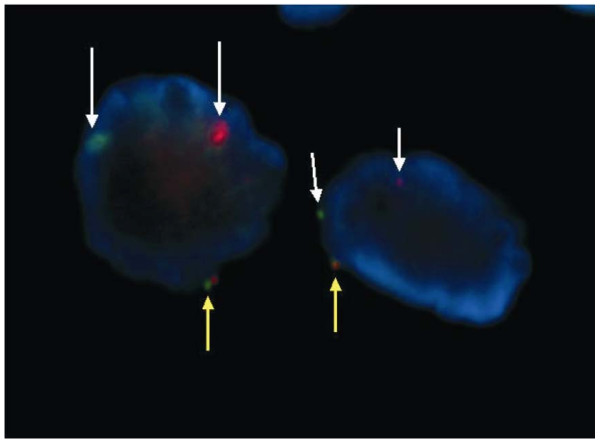
**FISH analysis of the high grade component of the submandibular lymph node biopsy specimen showed the presence of one fused signal (yellow arrows) and two split signal (white arrows) in single cell, indicating the presence of translocation of MYC**.

Ultrasound-guided biopsy of the abdominal mass revealed only high grade lymphoma infiltration with small round lymphoma cells carrying scanty cytoplasm, irregular nuclear outline and prominent nucleoli. PCR of DNA from both the submandibular lymph node and the abdominal lymph node biopsy showed presence of t(14;18). (Figure 3) FISH study on the high grade component of the submandibular lymph node confirmed the presence of *MYC *translocation (Figure 4).

## Comment

Our patient had intermediate-high risk atypical Burkitt's lymphoma transforming from underlying FL. This is based on the composite histology which showed the histological and molecular features of FL (follicles with aberrant expression of BCL2 protein and presence of IgH-BCL2 translocation by PCR), and features of atypical Burkitt's lymphoma in the interfollicular regions (starry sky appearance, high mitotic rate, 95% MIB1 positivity, CD10 expression and FISH evidence of MYC translocations in the small noncleaved lymphoma cells). Despite the absence of a history of low grade lymphoma in our patient, the evidence supporting transformation from an underlying FL includes the followings. First, in the submandibular lymph node, neoplastic follicles with aberrant expression of *BCL2 *were present amongst the high-grade lymphoma infiltration. Moreover, in the abdominal lymph node biopsy, which contained atypical Burkitt's lymphoma cells only, t(14;18) translocation was present, suggesting that the atypical Burkitt's lymphoma has transformed from an underlying FL which might have been clinically occult prior to this presentation. We believed the underlying FL cells acquired secondary MYC translocations during their somatic hypermutations. It is highly likely that additional cytogenetic abnormalities being acquired during this transformation process contributing to the histological progression and aggressive clinical course. We treated this patient with the Stanford V regimen as first line therapy, which yielded an unsatisfactory clinical response. Therefore we switched to IVAC, which comprised of a combination of different chemotherapeutic agents not seen by the patient in previous chemotherapies. Unfortunately, the disease was primarily refractory to chemotherapy.

Herein we reviewed the English literature of high-grade lymphoma transformation from underlying FL upon acquisition of MYC translocation as evidenced by metaphase cytogenetic, FISH or Southern hybridization. Patients with either de novo high-grade lymphoma or lymphoblastic leukemia, in which tumor cells possessed concomitant t(14;18) and c-MYC translocations [8,9], were not discussed as there was no evidence of underlying FL by history or histology.

In a review of the patients with histological transformations from underlying FL acquiring MYC translocations, two forms of presentation were identified. Majority developed aggressive Burkitt's transformation after a variable period from the diagnosis of underlying FL, ranging from 6 to 13 months [10-16]. Only three patients, in addition to the present case, were reported in the literature presenting with concomitant Burkitt's and FL. (Table 1) These three patients had composite histology showing features of both Burkitt's and FL in the same specimen, as in our case [17-19].

The acquisition of MYC translocation is reported in DLCL, lymphoblastic lymphoma and pre-B acute lymphoblastic leukemia transformation from FL, but only rarely in atypical Burkitt's lymphoma or Burkitt's leukemia transformation [4,5].

Unlike classical Burkitt's lymphoma in which extranodal presentation is most common, majority of these cases of atypical Burkitt's transformation had nodal presentation. All patients had a uniformly aggressive disease with poor survivals ranging from 4-13 months from the diagnosis of Burkitts' transformation. (Table 1) Moreover, the presence of combined MYC dysregulation, and BCL2 upregulation at diagnosis already predicted a very aggressive clinical course with rapid transformation into a high-grade lymphoma [4]. Furthermore, while MYC dysregulation associated with t(8;14) generally involved a different IgH allele from that involved by the underlying t(14;18), there are reports of rare transformation involving a complex chromosomal rearrangement in which both BCL2 and c-MYC are translocated to the same IgH allele [9]. Irrespective of the temporal relation of the histological progression and cytogenetic alterations, all the reported cases including our present case are very aggressive and highly resistant to chemotherapy. There is some evidence that radiotherapy may play an important role in disease control in this group of patients [20].

**Table 1:** Patients with composite histology of atypical Burkitt's and follicular lymphoma

Patient	This case	1	2	3
Age	58	77	52	44
Sex	M	M	F	M
Site and stage at initial presentation	Submand LN + abd LN, ascites Stage III	Generalized LN + BM Stage IV	LN Stage III	LN + BM Stage IV
Temporal sequence of transformation	Concomitant	Concomitant	Concomitant	Concomitant
Histology	Composite Lymphoma	Composite follicular + Burkitt's	Composite Follicular + Burkitt's	Composite Lymphoma
Cytogenetics at transformation	t(14;18) by PCR + MYC translocation by FISH	t(14;18) + t(8;14)	t(14;18) in follicular area t(14;18) + t(8;14) in transformed area	t(14;18) + t(8;14)
Treatment	Chemo	Chemo	Chemo → CR	Chemo → CR
Outcome	Primary refractory died 13 months after Dx	Primary refractory and died 4 months after diagnosis	Relapsed 3 months later with partial response to salvage chemo	CNS relapse 6 months later and died
Reference		17	18	19

Abbreviations: F: female; M: male; LN: lymph node; FISH: fluorescent in-situ hybridization; NA: not available; BM: bone marrow;CNS central nervous system;

Dx: diagnosis; CR: complete remission; PR: partial remission; NR: non-remission;

In summary, atypical Burkitt's transformation in our patient was illustrated by the composite histology, and FISH evidence of MYC translocation in addition to the presence of t(14;18). Moreover, additional MYC translocations in underlying FL may lead to high-grade lymphoma transformations with a wide range of histological subtypes in addition to atypical Burkitt's lymphoma/leukemia.

## Competing interests

The authors declare that they have no competing interests.

## Authors' contributions

YYH and CSC drafted the manuscript. FL and LPC carried out the immunohistochemical staining of tissue specimen and made the histological review. All authors read and approved the final manuscript.
